# Supplementary material for: Giant room temperature electrocaloric effect in a layered hybrid perovskite ferroelectric: [(CH3)2CHCH2NH3]2PbCl4
Source: Nat Commun. 2021 Sep 24;12:5502. doi: 10.1038/s41467-021-25644-x (PMC8463535; doi:10.1038/s41467-021-25644-x)
Supplement: Supplementary file 1 — Supplementary Information [file 41467_2021_25644_MOESM1_ESM.pdf]

Supplementary Information

Giant Room Temperature Electrocaloric Effect in a Layered Hybrid Perovskite Ferroelectric: $[(CH_3)_2CHCH_2NH_3]_2PbCl_4$

Xitao Liu,[1,2,*] Zhenyue Wu,[1,2,3] Tong Guan,[4] Haidong Jiang,[4] Peiqing Long,[1,2] Xiaoqi Li,[1,2] Chengmin Ji,[1,2] Shuang Chen,[4,*] Zhihua Sun,[1,2,3] and Junhua Luo[1,2,3,*]

[1]State Key Laboratory of Structural Chemistry, Fujian Institute of Research on the Structure of Matter, Chinese Academy of Sciences, Fuzhou, 350002, China

[2]Fujian Science & Technology Innovation Laboratory for Optoelectronic Information of China, Fuzhou, Fujian, 350108, China

[3]University of Chinese Academy of Sciences, Chinese Academy of Sciences, Beijing, 100039, China

[4]Kuang Yaming Honors School and Institute for Brain Sciences, Nanjing University, Nanjing, Jiangsu 210023, China

Supplementary Methods

Measurements. Powder XRD data of ground powder from grown single crystals were measured in the range from 5° to 50° with a scan step of 0.02°/0.4 s on a powder X-ray diffractometer (MiniFlex 600, Rigaku Co., Tokyo, Japan) at room temperature.Absorption spectrum was implemented on a Perkin-Elmer Lambda 900 UV-Vis-NIR spectrum photometer at room temperature with $BaSO_4$ used as the 100% reference. The band gap was calculated according to the Kubelka–Munk equation: $\alpha/S = (1–R)^2/2R$.

Quantitative prediction of EC performance. Learning from the experimental XRD results of Figure 1 in the main text, the difference between the monoclinic phase at 285 K (corresponding to the ferroelectric phase) and the orthorhombic phase at 343 K (corresponding to the paraelectric phase) of our studied hybrid perovskite $(iBA)_2PbCl_4$, can be located to help us recognize degrees of freedom for switching this ferroelectric-paraelectric phase transition. We find that the molecular motions of iBA^+ cations, including their rotation around the layer stacking direction and the flipping of $-CH_2-NH_3$ groups, are driving force to switch this phase transition. At the same time, the inorganic $PbCl_6$ octahedra would undergo distortion. The climbing-image nudged elastic band (CI-NEB) calculations can be employed to collect the information of this phase transition pathway that connects the initial ferroelectric phase (the left one) to the final symmetry-corresponding ferroelectric phase (the right one) via the paraelectric phase (the middle one) as the transition state in Figure S14a. For our studied hybrid perovskite, its phase transition is more complex than pure inorganic perovskites because of the existence of organic cations. Therefore, one reaction coordinate λ in Supplementary Fig. 14 is used to synergistically correlate all the degrees of freedom mentioned above to span this phase transition. Our treatment is quite simple and different from the soft mode theory used in pure inorganic perovskites.[S1] In the soft mode theory, the phase transiton free energy can be expressed by a series of order parameters corresponding to certain soft phonon modes.[S1] Futher learning from Figure S14b, the polarization of hybrid perovskite ferroelectric $(iBA)_2PbCl_4$ can also be derived as a function of the reaction coordinate λ during this phase transition. Thus, the polarization can be written as:

$$P = P(\lambda) \quad (1).$$

With reference to the Landau-Ginzburg theory for improper ferroelectrics[S1, S2] and futher in consideration of the complex phase transition process of our studied hybrid perovskite $(iBA)_2PbCl_4$, its thermodynamic energy (G) can be simply expanded as follows:

$$G = G_0 + \mu\lambda^2 + \nu\lambda^4 + \kappa\lambda^6 - EP(\lambda) \quad (2)$$

where G_0 indicates its zero point of the thermodynamic energy; P represents polarization; E is external electric field; and μ, ν, and κ are different-order expansion parameters. Then learning from Figure S14a and S14b, the final fitting expression of free energy is obtained to further calculate the entropy change and temperature change of our studied system to discuss its electrocaloric properties.

According to the classical thermodynamic relations, the entropy change per mass of $(iBA)_2PbCl_4$ can be calculated by:

$$\Delta S = \left(\frac{\partial G}{\partial T}\right)_E \quad (3).$$

Finally, the entropy change during the electrocaloric cycle under the isothermal condition and under the applied electric field can be derived as:

$$\Delta S_{\mathrm{EC}} = \Delta S(T,\ E = E_{\mathrm{app}}) - \Delta S(T,\ E = 0) \qquad (4).$$

And the temperature change during the adiabatic process can be approximately obtained as:

$$\Delta T_{\mathrm{EC}} \approx - \frac{T\Delta S_{\mathrm{EC}}}{c_p} \qquad (5)$$

in which T is the working temperature while C_p is specific heat fitted according to the experimental DSC curves in Figure S8.

As shown in Figure 5a and 5b, our computational isothermal entropy changes and adiabatic temperature changes indicate that the EC performance of $(iBA)_2PbCl_4$ is almost one order magnitude larger than those of the traditional ferroelectrics, with an entropy change ΔS of about 22.5 J/kg/K and an adiabatic temperature change ΔT of about 7.9 K at room temperature under the applied electric field of 23.2 kV/cm, which confirms the results calculated based on the classical Maxwell thermodynamic relation in the main text. The isothermal entropy change ΔS and adiabatic temperature change ΔT under the applied electric field of 15.2 kV/cm still reach 20.5 J/kg/K and 7.1 K respectively, which are relatively larger than those calculated based on the Maxwell theory.

Computational details for the first-principles calculations. All the first-principles calculations here are performed in the Vienna Ab initio Simulation Package (VASP, Version 5.4.4). The PBE-D3 functional is used within the framework of the generalized gradient approximation (GGA). The projector augmented wave (PAW) potentials with an energy cut-off of 500 eV is implemented to realize a good convergence. The Gaussian smearing with the width of 0.05 eV is used for all calculations. For their geometry optimization, the total energy change is set to be less than 10^{-5} eV, and the magnitude of the largest force acting on the atoms is set to be less than 0.02 eV/Å. The automatic *k*-point mesh of 3×1×3 is used for all the structures. In order to verify the phase transition mechanism of our studied system, the CI-NEB calculations are performed to find possible reaction pathway with the total energy set to be less than 10^{-5} eV and the magnitude of the largest force acting on the atoms set to be less than 0.09 eV/Å. Finally, the spontaneous polarization of each structure along the obtained phase transition pathway can be calculated by using the Berry phase method. In order to obtain accurate spontaneous polarization values, more accurate single-point calculations are performed with the energy converge set to 10^{-7} eV and a denser automatic *k*-point mesh of 6×2×6.

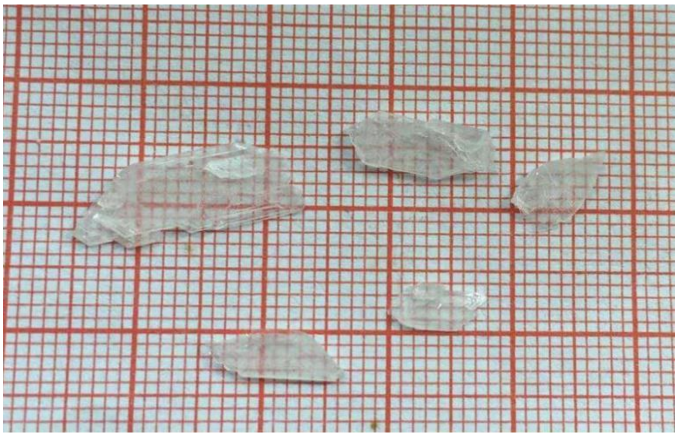

Supplementary figure 1. The grown single crystals of $[(CH_3)_2CHCH_2NH_3]_2PbCl_4$.

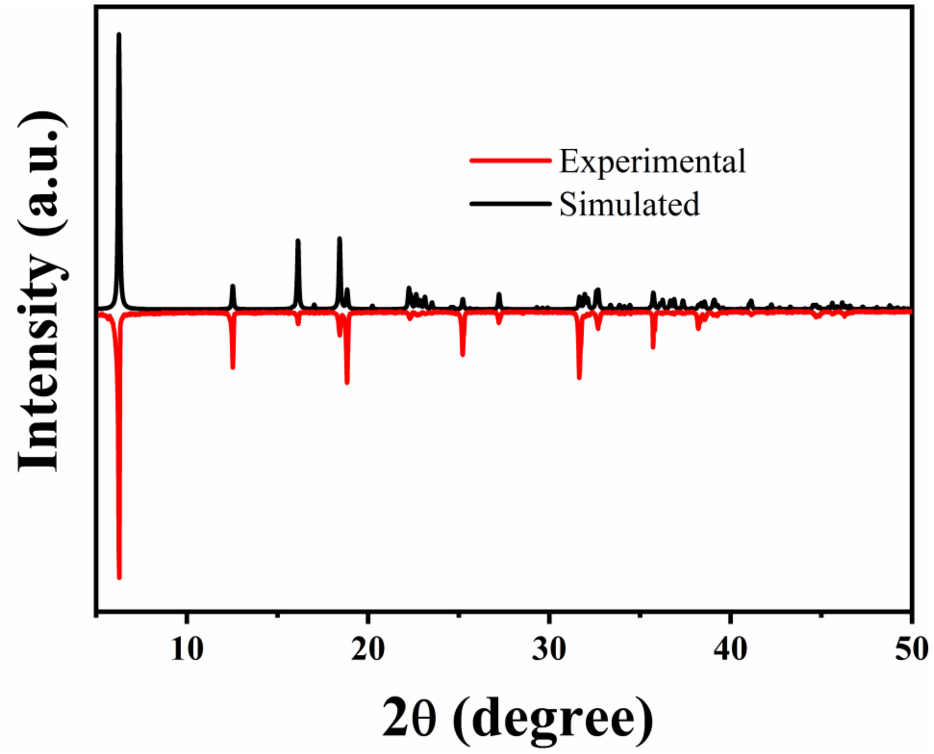

Supplementary figure 2. Experiment and simulated powder X-ray diffraction patterns of **1**.

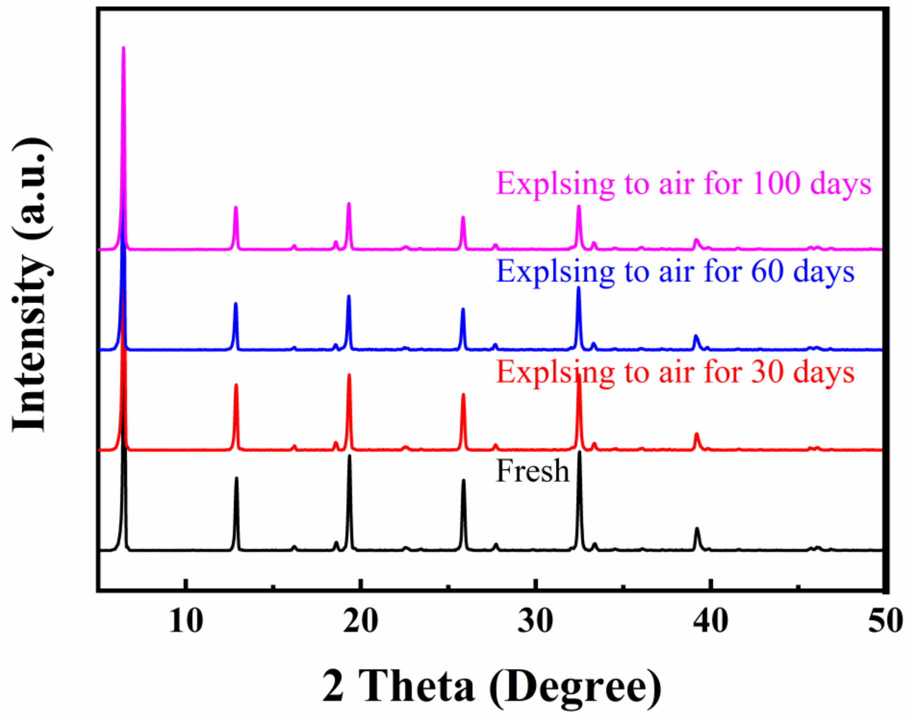

Supplementary figure 3. Powder X-ray diffractions patterns of **1** in the different test conditions.

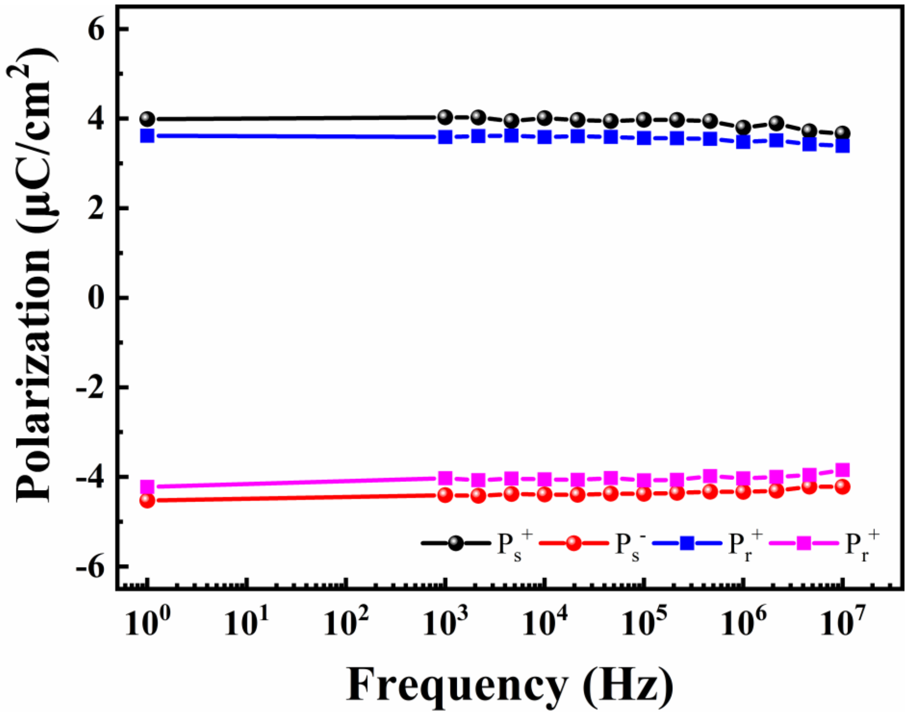

Supplementary figure 4. V Figure S4. Variation of polarization versus number of switching cycles of **1**.

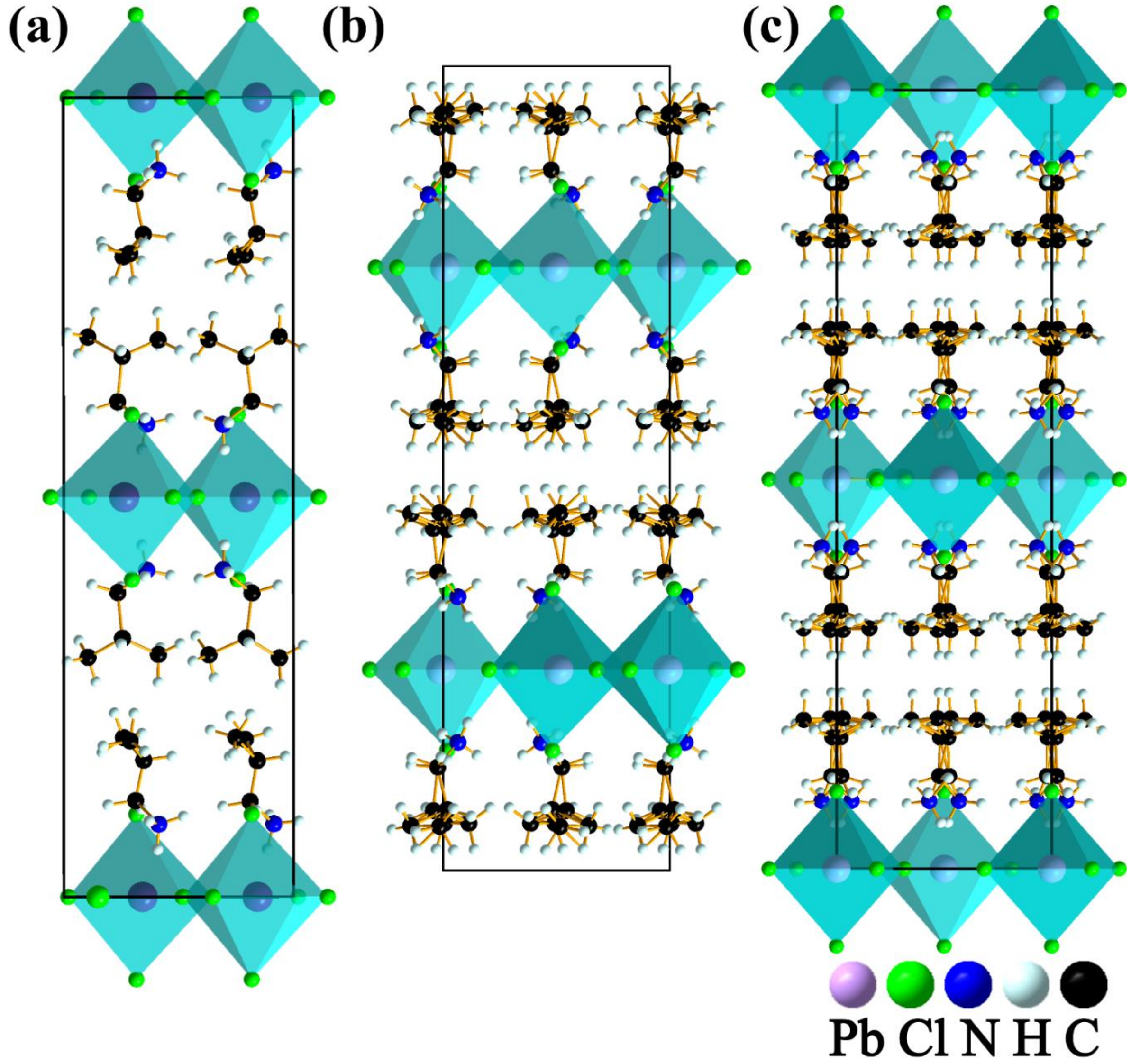

Supplementary figure 5. Crystal structures of **1** at different phases. (a) Packing diagram at at 285 K; (b) Packing diagram at 318 K; (c) Packing structure at 343 K.

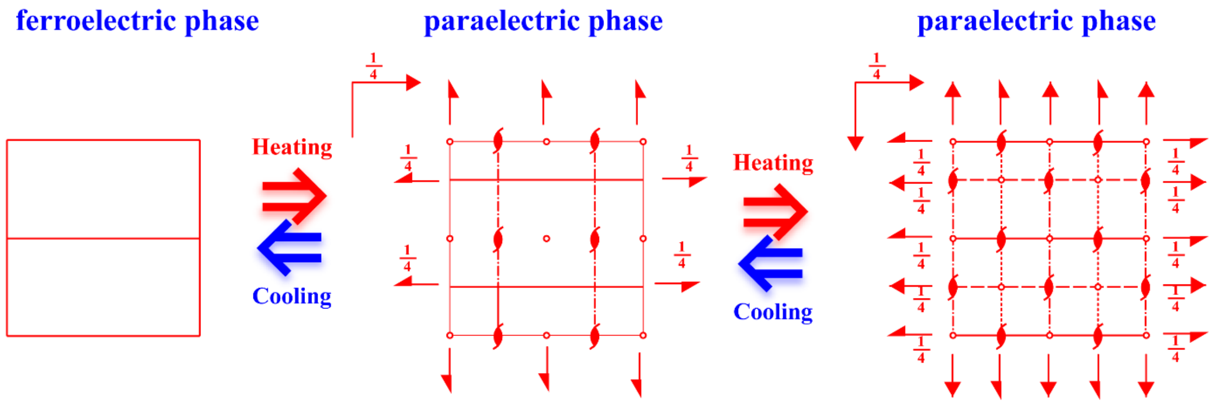

Supplementary figure 6. Symmetry breaking occurs of **1** from the ferroelectric phase (LTP) to paraelectric phase (ITP) and then to the paraelectric phase (HTP) with a Shuvalov's symbol of mmmFm. The symmetry operation elements from 8 (E, C_2, C_2', $C2''$, i, σ_h, σ_v, σ_d) at the HTP to 8 (E, C_2, C_2', $C2''$, i, σ_h, σ_v, σ_d) at the ITP and finally to 2 (E, σ_h).

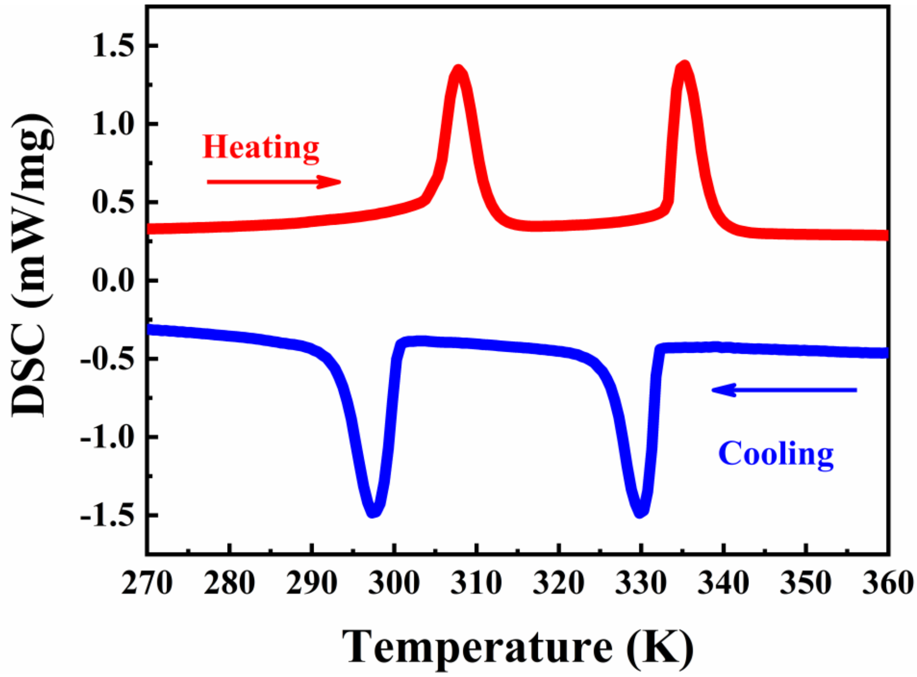

Supplementary figure 7. DSC curves in the heating and coling runs of **1**.

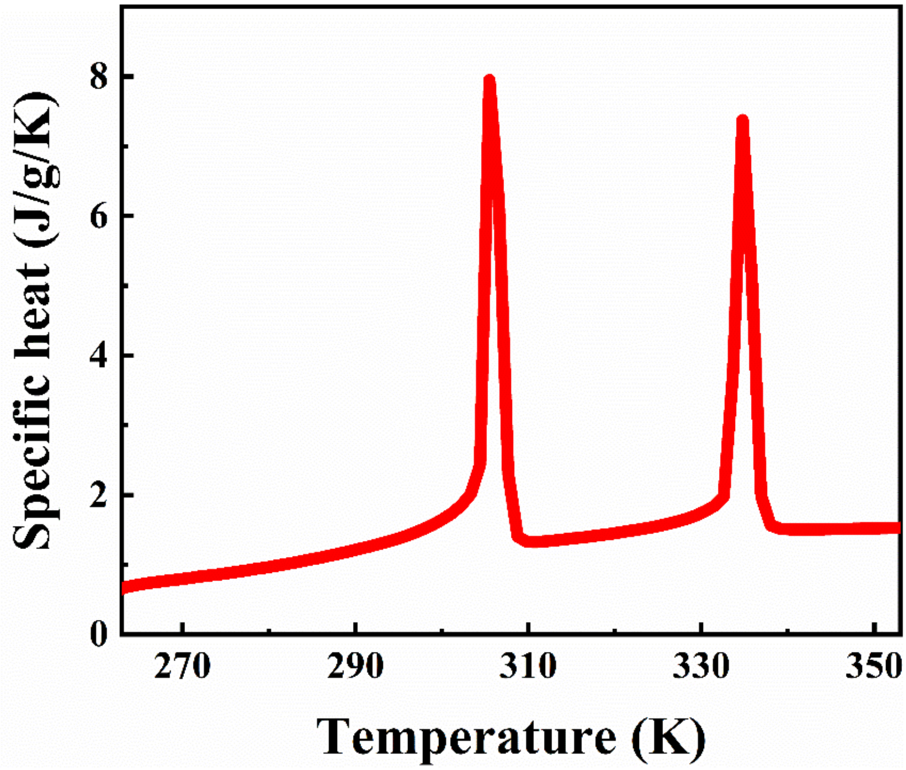

Supplementary figure 8. Heat capacity-temperature curve of **1**.

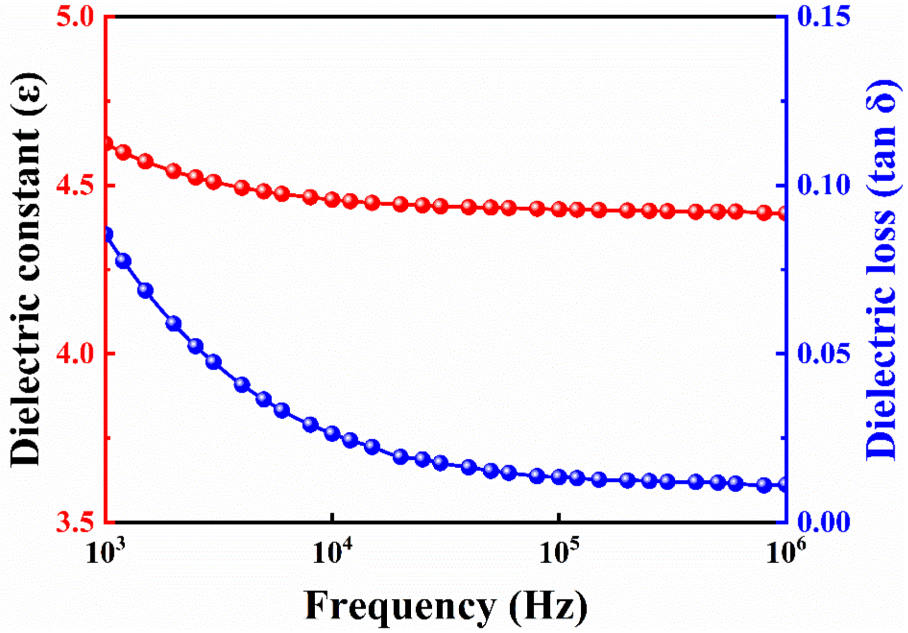

Supplementary figure 9. Frequency-dependent dielectric properties of **1.**

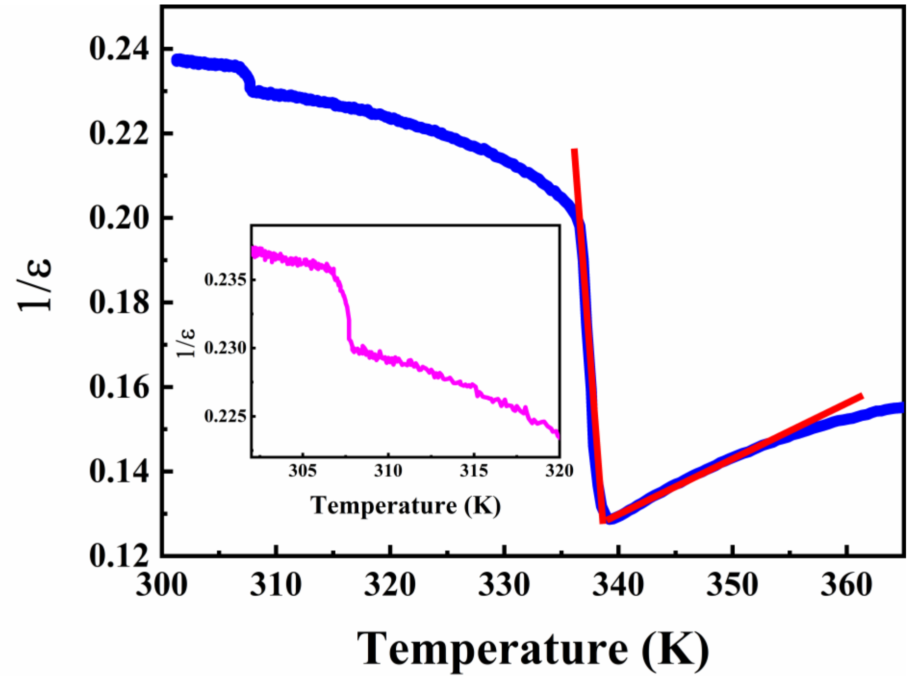

Supplementary figure 10. The fitting to Curie-Weiss law of dielectric anomalies at 500 kHz.

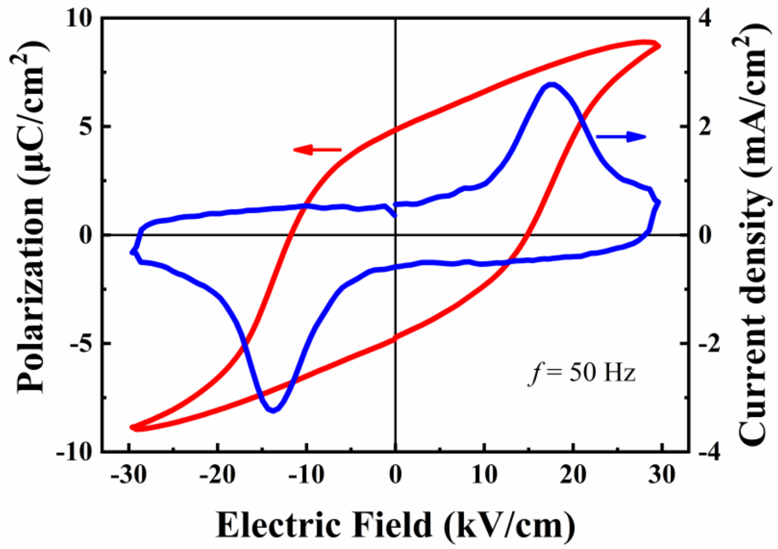

Supplementary figure 11. P-E hysteresis loops measured along the ***a***-axis at 293 K.

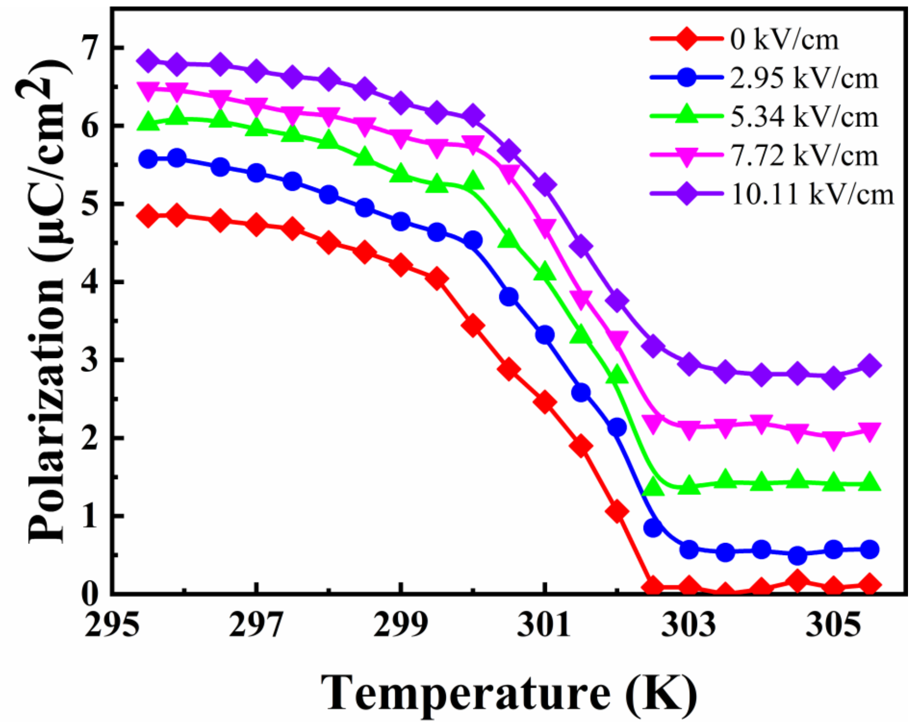

Supplementary figure 12. Temperature-dependent polarization at low electric fields.

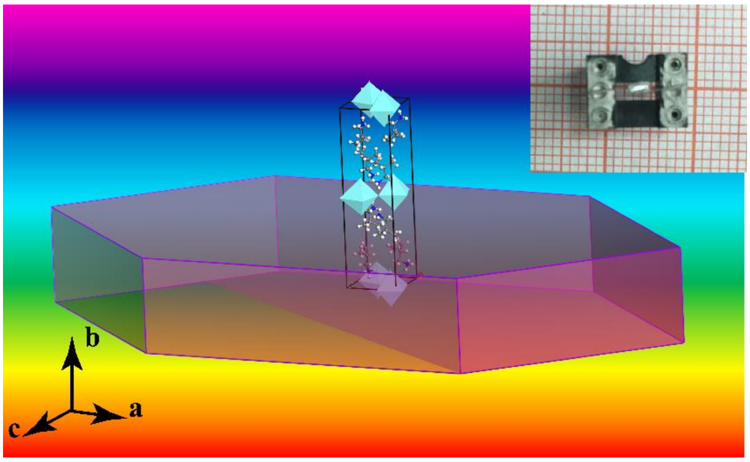

Supplementary figure 13. Crystal morphology of grown crystals. Inset: fabricated ferroelectric capacitor.

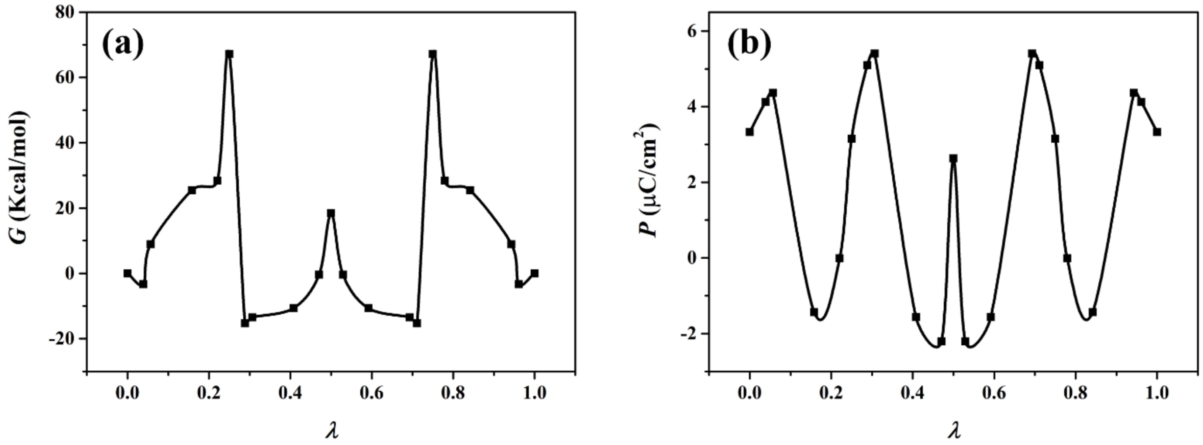

Supplementary figure 14. (a) Variation of free energy (G) and (a) variation of polarization (P) with reaction coordinate (λ) for ferroelectric-paraelectric-ferroelectric phase transiton of hybrid peroskite $(iBA)_2PbCl_4$ based on the CI-NEB calculations.

Supplementary table 1. Crystal data for 1 collected at 285 K, 318 K and 343 K.

Empirical formula	$[(CH_3)_2CHCH_2NH_3]_2PbCl_4$	$[(CH_3)_2CHCH_2NH_3]_2PbCl_4$	$[(CH_3)_2CHCH_2NH_3]_2PbCl_4$
Formula weight	497.28	497.28	497.28
Temperature (*K*)	285	298	343
Space group	*Pm*	*Pnma*	*Cmce*
Cell parameters	***a*** = 7.9019(3)Å<br>***b*** = 27.4789(15)Å<br>***c*** = 7.9019(3) Å<br>***α*** *= 90°*<br>***β*** *= 90.0000(10)°*<br>***γ*** *= 90°*	***a*** = 7.8750(9) Å<br>***b*** = 28.227(3) Å<br>***c*** = 7.9651(8) Å<br>***α*** *= 90°*<br>***β*** *=90°*<br>***γ*** *= 90°*	***a*** = 28.5413(18) Å<br>***b*** = 7.8912(4) Å<br>***c*** = 7.8938(4) Å<br>***α*** *= 90°*<br>***β*** *= 90°*<br>***γ*** *= 90°*
V (Å^3)	1715.78(13)	1770.6(3)	1777.88(17)
Z, ρ_{cal}. (g/cm^3)	4, 1.925	4, 1.866	4, 1.858
F(000)	944.0	944.0	944.0
Radiation λ (Mo Kα) (Å)	0.71073	0.71073	0.71073
2Theta range (°)	4.446 to 55.06	6.702 to 50	7.44 to 55.006
Limiting indices	-10 ≤ h ≤ 10<br>-32 ≤ k ≤ 35<br>-10 ≤ l ≤ 10	-9 ≤ h ≤ 9<br>-33 ≤ k ≤ 30<br>-9 ≤ l ≤ 7	-36 ≤ h ≤ 36<br>-10 ≤ k ≤ 10<br>-10 ≤ l ≤ 10
Reflections collected /unique	14368/ 6378	7470/ 1519	11665/ 1051
Data/restraints/parameter	6378/2/301	1519/140/108	1051/32/65
GOF	0.982	1.766	1.051
Final *R* indices [$I > 2\sigma(I)$][a]	R_1 = 0.0441<br>wR_2 = 0.0986	$R_1 = 0.1316$<br>$wR_2 = 0.3703$	$R_1 = 0.0159$<br>$wR_2 = 0.0345$
R indices (all data)	R_1 = 0.0515<br>wR_2 = 0.1024	$R_1 = 0.1353$<br>$wR_2 = 0.3739$	$R_1 = 0.0261$<br>$wR_2 = 0.0382$
Largest diff. peak and hole, e/Å^{-3}	4.79/-2.89	10.83/-3.53	0.25/-0.22
Flack parameter	0.075(6)	--	--

[a]$R_1 = \Sigma||F_o| - |F_c||/\Sigma|F_o|$, $wR_2 = [\Sigma w(F_o^2 - F_c^2)^2/\Sigma(F_o^2)^2]^{1/2}$

Supplementary references

S1. Young, J. Stroppa, A. Picozzi, S. Rondinelli, J. M. Anharmonic lattice interactions in improper ferroelectrics for multiferroic design. *J. Phys. Condens. Matter.* **27**, 283202 (2015).

S2. Holakovský, J. A New Type of the Ferroelectric Phase Transition. *Phys. Status Solidi B* **56**, 615-619 (1973).
