# Supplementary material for: Giant room temperature electrocaloric effect in a layered hybrid perovskite ferroelectric: [(CH3)2CHCH2NH3]2PbCl4
Source: Nat Commun. 2021 Sep 24;12:5502. doi: 10.1038/s41467-021-25644-x (PMC8463535; doi:10.1038/s41467-021-25644-x)
Supplement: Supplementary file 3 — Description of Additional Supplementary Files [file 41467_2021_25644_MOESM3_ESM.pdf]

Description of Additional Supplementary Files

File Name: Supplementary Data 1

Description: The crystallographic structure data at 285 K

File Name: Supplementary Data 2

Description: The crystallographic structure data at 318 K

File Name: Supplementary Data 3

Description: The crystallographic structure data at 343 K
